# Spontaneous firing activity in climbing fiber is critical for a realistic bi-hemispherical cerebellar neuronal network during robot control

**DOI:** 10.1186/1471-2202-15-S1-P142

**Published:** 2014-07-21

**Authors:** Ruben-Dario Pinzon-Morales, Yutaka Hirata

**Affiliations:** 1 Department of Computer Science, Chubu University Graduate School of Engineering, Kasugai, 487-8501, Japan

## Introduction

The cerebellum plays a crucial role in short-term motor learning, sensory-motor transformations, and cognitive functioning [1,2]. In motor control and learning, the cerebellum adaptively accounts for development, aging, injuries all that may cause asymmetries in neuromuscular and optical properties [3]. Previously we have configured a physio-anatomically inspired bi-hemispherical cerebellar neuronal network (biCNN), and demonstrated that the biCNN can govern an unstable 2-wheeled balancing robot under asymmetrical perturbation conditions [4]. Exalted by observations of bilateral plasticity during unilateral learning paradigms [3,5,6], right and left hemispheres of the cerebellum were separately modeled in the biCNN. Each hemisphere included granular (Gr), Golgi (Go), basket/stellate (Ba/St), and Purkinje (Pk) cells. Excitatory inputs to the biCNN carried by mossy fibers (mfs) provided the desired motion trajectories. Inhibitory feedback loop between Gr and Go, and feed-forward inhibitory loop between Ba/St and Pk were also included. The teaching signal to the Pk via climbing fibers (cf) was implemented to include the very low spontaneous spike activity (~ 1 spikes/s) observed in this fiber [5,6]. A proportional and differential (PD) controller sharing the same mfs inputs was introduced to represent the non-cerebellar input to the vestibular nucleus (Vn). The Vn computed the arithmetic difference between the PD and the Pk cells output and produced the motor command to the robot. The error signal originated from the concurrent PD controller was split into two by using half-wave rectifiers. The negative and positive waves, akin the forward and backward motion errors, were fed into left and right hemisphere, respectively after adding a DC value to mimic the climbing fiber spontaneous firing. A simple learning rule that adjusts the efficacy of the Gr-Pk synapses was included to mimic long-term depression and potentiation discovered at this synapse *in vitro* and *in vivo*. Currently a further refinement of the biCNN was made to study the cerebellar mechanisms during asymmetrical motor control.

## Methods

Firstly, an inhibitory input to Go cells via Lugaro (Lu) cells was implemented [8]. Secondly, the number of neuron models in each hemisphere was increased (from 1.500 to 20.000) to better reflect the ratio of neurons in the real cerebellum [1]. Each hemisphere comprised 128 mf, 9956 Gr, 15 Go, 300 Ba/St, 3 Lu, and 30 Pk cells. Thirdly, mutual inhibitory synapses between Pk cell and Ba/St was included, and finally a simple learning rule governing the Pk-Vn synaptic plasticity was implemented [1]. The robot was commanded to follow a sinusoidal (f = 0.25 Hz) wheel motion for 200 cycles. At cycle 100, an asymmetrical perturbation was added.

## Results

Results showed that asymmetrical perturbations to the robot were successfully handled by the biCNN, and that the spontaneous activity in the cf was critical for balancing the contribution of the cerebellar hemispheres to the Vn. Disabling the spontaneous activity endangered the biCNN output during the asymmetrical robot control scenario.

## Conclusions

In our robot control framework, the cf input carrying spontaneous activity drove the learning in the two hemispheres of the biCNN required to control the unstable robot during asymmetrical perturbations.
